# The “authentic subjective experience” of memory in Alzheimer’s disease

**DOI:** 10.1515/tnsci-2020-0123

**Published:** 2020-06-03

**Authors:** Mohamad El Haj, Ahmed A. Moustafa, Jean Roche, Florence Pasquier, Dimitrios Kapogiannis, Karim Gallouj, Pascal Antoine

**Affiliations:** Univ Nantes, Laboratoire de psychologie des Pays de la Loire, LPPL, EA 4638, F-44000 Nantes, France; Unité de Gériatrie, Centre Hospitalier de Tourcoing, Tourcoing, France; Institut Universitaire de France, Paris, France; School of Social Sciences and Psychology & Marcs Institute for Brain and Behaviour, Western Sydney University, Sydney, Australia; Department of Human Anatomy and Physiology, the Faculty of Health Sciences, University of Johannesburg, Johannesburg, South Africa; CHU de Lille, Unité de Psychogériatrie, Pôle de gérontologie, 59037 Lille, France; University Lille Nord de France UDSL and Memory Clinic, CHU, Lille, France; Laboratory of Neurosciences, National Institute on Aging, Baltimore, MD, USA; Univ. Lille, CNRS, CHU Lille, UMR 9193 – SCALab – Sciences Cognitives et Sciences Affectives, F-59000 Lille, France

**Keywords:** Alzheimer’s disease, autobiographical memory, consciousness, subjective experience

## Abstract

Most research has mainly focused on the decline of the subjective experience in Alzheimer’s disease (AD). However, few attempts have been made to evaluate whether subjective experience may be maintained in AD. In this narrative review, we attempt to provide a positive view, according to which patients with AD can enjoy, to some extent, subjective experience during memory retrieval. Memory and expression difficulties (e.g., aphasia) limit the ability of patients with AD to describe their memories, resulting in a little specificity of reported memories. However, according to the “authentic subjective experience” view, we propose in this study that the ability to mentally relive these memories could be preserved in the patients. By proposing the authentic subjective experience view, we attempt to provide an alternative view to the general consideration that the patients suffer a diminished subjective experience. This view can contribute to a larger clinical framework that gives a positive meaning to the subjective experience of patients with AD. Furthermore, several clinical and empirical implications can be drawn from the authentic subjective experience view, including the possibility to evaluate behavioral correlates of the subjective experience in AD.

## 1 Introduction

Memory decline is widely considered as the cognitive hallmark of typical Alzheimer's disease (AD) [1]. Psychological research has been mainly concerned with the diminished ability of the patients to retrieve and relive past experiences [2]. In this study, we argue that patients with AD may still be able to enjoy an authentic subjective experience of memory, that is, they can experience an authentic sense of reliving during memory retrieval. At the clinical level, this view is worth considering because by defending the view that patients with AD possess an “authentic subjective experience” of the past, we attempt to offer a positive view of their potential to introspect into their memory functioning and cognitive performance in general.

This review begins by providing definitions of basic terms (e.g., subjective experience and lack of insight). We then emphasize empirical support to the authentic subjective experience view that we defend in this study. Finally, we discuss the implications of the view, as well as venues for future empirical research. Note that, while this review is narrative, we also performed a literature search for papers dealing with the subjective experience of autobiographical memory in AD. In this literature search, we used MEDLINE (via PubMed) and Google Scholar databases using a combination of the following search terms and key words: Alzheimer’s disease, anosognosia, autobiographical memory, autonoetic consciousness, consciousness, denial, and insight. The search resulted in 464 papers, and we screened these papers to identify those dealing with the subjective experience of autobiographical memory in patients with AD, resulting in the below-discussed papers.

### 1.1 Basic definitions

The subjective experience of episodic memory can be defined with reference to “subjective experience” as proposed by Tulving [3] who associates episodic recall with a feeling of re-experiencing (i.e., autonoetic consciousness), which refers to the ability to mentally project one's existence into a subjective time. Impairment in mental time travel can be associated with memory distortions in AD. According to the Temporal Consciousness Model [4], patients with confabulations (including those with AD) have a disturbed sense of chronology, such that they are able to retrieve the content of events but not their order of occurrence. Consequently, they misattribute the features of events that occurred at one time to other events that occurred at another time. Regardless of memory, the subjective experience in patients with AD is a complex and non-unitary phenomenon [5,6,7,8]. The same thing can be said for the concept of consciousness of general. Although consciousness can be defined as the awareness of the self and the environment [9], it also includes several phenomenological components (e.g., the subjective experience) and cognitive aspects (e.g., perception, affect, and control).

The literature proposes several terms (e.g., “denial,” “lack of insight,” and “anosognosia”) to describe the decline of the subjective experience in AD. The terms “denial” and “lack of insight” are typically used to describe a lack of introspective ability and metacognitive functioning, whereas the term “anosognosia” is typically used to describe a failure to acknowledge a particular cognitive deficit [10,11]. Theoretical accounts on the mechanism of anosognosia in AD have associated a lack of insight with a disconnection of the “conscious awareness system.” The conscious awareness system is a cognitive system that detects the changes in cognitive functioning based on the inputs from various brain regions [12,13]. Any disconnection between the conscious awareness system and the changes in a given cognitive domain (e.g., memory) results in a lack of awareness within that cognitive domain. A similar suggestion was made by the Cognitive Awareness Model [14,15]. This model is composed of components receiving information from autobiographical memory and comparator components monitoring performances. All these components provide an output that feeds into awareness about the actual performances. According to the Cognitive Awareness Model, patients with unawareness of memory deficits have difficulties to encode those failures into memory, relying on outdated representations of their memory functioning. Research on anosognosia emphasizes the ability of patients with AD to estimate their own memory functioning and introspect into their own subjective experience during recall. While this research explains how the patients estimate and experience their own memory functioning, it runs the risk of denying the patients any level of awareness of their memories (e.g., “lack of awareness” and “anosognosia”). However, the concept of awareness in AD, at least the subjective experience of memory, is not necessarily negative since patients may be able to enjoy an authentic subjective experience of the past. We here discuss the research demonstrating how patients with AD can enjoy an authentic subjective experience of memory.

## 2 The authentic subjective experience view

In a previous study, we investigated whether patients with AD may report a high subjective experience of autobiographical memories [16]. We asked patients with mild AD and the control participants to retrieve three autobiographical memories. For each memory, the participants were invited to rate its subjective characteristics on items assessing travel in time, remembering, reliving, realness, visual imagery, auditory imagery, emotion, and importance. Besides this subjective evaluation, we analyzed the specificity of the retrieved memories (i.e., whether memories described general events or specific events situated in time and space). AD participants showed lower general autobiographical recall than the controls, and poorer reliving, travel in time, remembering, realness, visual imagery, auditory imagery, language, rehearsal, and spatial detail – a decrease that was especially pronounced for visual imagery. Yet, AD participants showed high rating for emotion and importance. Critically, a discrepancy (i.e., higher levels of subjective reliving than objective recall) was observed in patients with AD but not in the control participants. In other words, despite a compromise in their (objective) autobiographical performance, patients with AD attributed a high value to their subjective experience, especially to emotion and importance of memory. This discrepancy could be due to an overestimation and alternatively to a high consciousness experience during which patients with mild AD possess authentic subjective experiences of the past. This interpretation is further supported by a study demonstrating that despite a compromise in some subjective features of recall (e.g., in the ability to create a visual image of the retrieved event), other subjective features (i.e., emotion and importance of the recall) can be fairly experienced by patients with mild AD [17].

We propose the authentic subjective experience view to explain the discrepancy between subjective reliving and objective recall in patients with AD. This is different from the anosognosia account. While the anosognosia account also deals with the subjective experience of remembering, this account attributes the failure to introspect into one’s own performance to a discrepancy between the expected and actual inputs. Our view emphasizes how patients with AD, and patients with amnesia in general [18], attribute a high value to their sense of reliving as a result of their authentic ability to experience the retrieved event rather than as a result of their failures to experience it.

**Figure 1:**
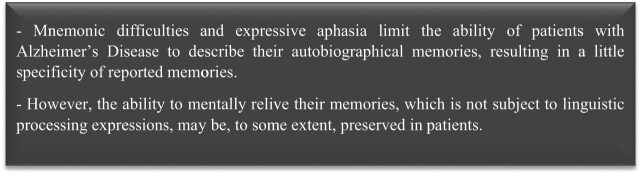
The “authentic subjective experience” account.

As illustrated in Figure 1, the authentic subjective experience view is summarized as follows: when asked to retrieve and report autobiographical memories, patients with AD may struggle to describe them, probably due to difficulties in both retrieving and expressing their memories. However, these patients may be able to enjoy an authentic subjective experience of their memories (with corresponding emotions), as this experience does not require linguistic processing. The difficulty to describe specific autobiographical events in AD is in agreement with the large body of research demonstrating a diminished ability of the patients to retrieve and report specific autobiographical memories [19,20,21,22,23,24,25,26,27,28,29,30,31,32]. In this body of research, the patients are typically asked to describe verbally the retrieved memories. This verbal description may limit the patients’ ability to detail retrieved memories, which is supported by a research demonstrating significant correlations between autobiographical retrieval and verbal fluency [33]. This suggestion can be also supported by the fact that AD affects several aspects of language, typically starting with semantics before proceeding to syntax and phonology and eventually leads to frank aphasia, a multimodal clinically significant disorder of understanding and producing language [34]. Moreover, these linguistic difficulties may limit the ability of patients with AD to describe memories and their subjective experience. The authentic subjective experience view can be supported by a research on normal aging, demonstrating that narrative style can hinder the ability of healthy older adults to retrieve specific memories [35,36]. The authentic subjective experience view can also be supported by a study demonstrating that narrative abilities influence the ability of patients with amnesia to describe pictures and imagined scenarios [37].

Several theoretical and clinical implications can be drawn from the authentic subjective experience view. At the theoretical level, this view contributes to the understanding of consciousness in patients with AD, especially to the understanding of how these patients relive and have a sense of ownership of memories. The authentic subjective experience view focuses on the ability of the patients to travel back in time to re-experience the past. The authentic subjective experience view can be also considered as an attempt to demonstrate how patients with AD can benefit from an authentic subjective experience when retrieving personal memories.

Another theoretical implication of the authentic subjective experience view is that it emphasizes the heterogeneity of consciousness experience in AD. Research on the subjective experience of memory in AD has typically evaluated different forms of this experience, such as sense of self [38], metamemory [39], anosognosia [15], or even denial [40], suggesting a heterogeneity of the consciousness experience in AD. The authentic subjective experience view extends this research by suggesting how, despite a compromise of anosognosia, other components can be relatively spared in AD (e.g., the subjective experience of autobiographical memory). It is noteworthy that this mixed picture can also be observed for specific consciousness experiences in AD. For instance, individual differences among AD patients in anosognosia can be observed depending on the assessment tool, the stage of dementia, and the type of memory or other cognitive function in question.

Regarding normal aging, the subjective experience during autobiographical retrieval was evaluated by Piolino et al. [41] who invited healthy older adults to retrieve recent and remote autobiographical memories. After memory retrieval, the participants had to provide a “Remember” response, if they were able to recover a specific event with its encoding context, and a “Know” response, if they just knew this event had happened to them but could not recall any contextual detail. Hence, Remember responses referred to autonoetic consciousness, whereas Know responses referred to noetic consciousness. The results demonstrated increased Know responses with age for recent but not for remote memories, supporting the view that autonoetic consciousness is relatively better preserved in healthy older adults with respect to the distant past than with respect to the more recent past. Note that, using the Remember/Know paradigm, a research has demonstrated increased Remember responses in patients with AD [42,43], suggesting a decreased autonoetic consciousness in AD. However, this research has been concerned with memory for simple stimuli (e.g., words and pictures) rather than with autobiographical memory.

One theoretical implication of the authentic subjective experience view is its potential effect on the patients’ sense of ownership of past experiences (i.e., the feeling that these experiences belong to them). This speculation can be compared with that of James [44] who distinguished between the self as an agent (the I) and the self as an object of reflection (the Me). According to James [44], personal ownership, as dependent on the “I”, is accompanied by a subjective experience imbued with a sense of warmth and intimacy. This colorful reliving can be associated with the subjective experience of the past in patients with AD, an assumption supported by our previous research demonstrating that these patients attribute high personal importance to their recall [16,17].

Altogether, the authentic subjective experience view can be considered as the outcome of the interaction between representational content (i.e., the retrieved memory), conscious apprehension, and sense of ownership resulting into the warmth and intimacy that characterizes the subjective experience of the past.

## 3 Toward a positive view of the consciousness experience in patients

As illustrated in the previous section, one implication of the authentic subjective experience view is that whereas some aspects of the consciousness experience can be affected by AD (e.g., anosognosia), other aspects (e.g., the ability to relive autobiographical memories) can be fairly experienced by patients with AD. At the clinical level, this implication is important, as it emphasizes how patients with AD can generate a subjective experience. Another implication is that by enjoying a fair subjective experience of autobiographical memories, patients with AD may be able to experience a rich sense of self and identity unlike what is generally assumed. This speculation can be supported by a research demonstrating an intimate relationship between the autobiographical performance and the sense of self in AD [33,45].

The authentic subjective experience view is also an attempt to alleviate the social stigma that can be associated with the disease. Needless to say, AD is mainly associated with dysfunction and poor cognitive abilities, and the social stigma tends to depict the disease as the “death sentence” and the patients as “empty shells” who experience a “death of the mind” [46,47]. The authentic subjective experience view can be considered as an alternative that identifies probably partially spared components of consciousness (i.e., the subjective experience of past) in patients with AD. We hope that this positive signal may prompt other researchers and clinicians to investigate, or at least consider, remaining intact cognitive functions in AD. We also hope that this view contributes to care models that give positive meaning to the consciousness experience of the patients and their ability to introspect into their cognitive functioning and, consequently, into their ability to judge, think, plan, and decide.

### 3.1 Perspectives: behavioral evaluation of the authentic subjective experience

One major challenge for autobiographical retrieval in AD, besides mnemonic difficulties, is aphasia. The degree to which an authentic subjective experience occurs may be partially beyond language-based description. Nevertheless, the validity of this view can be subjected to rigorous research to delineate the behavioral correlates of autobiographical memory in AD using non-linguistic means. This future research may be based on facial expression techniques. Due to advances in the technology allowing a reliable analysis of facial expressions, there is a burgeoning interest in facial expressions that can be activated during autobiographical retrieval [48,49]. A study reported that emotional memories trigger the corresponding basic facial expressions (i.e., high happy facial expressions for happy memories and sad facial expressions for sad memories) [50]. Another study reported higher happy emotional facial expressions during imagining the future than during remembering the past [51]. It would be of interest to evaluate whether emotional facial expressions are triggered by retrieval of autobiographical memories in AD, as such expressions would provide behavioral evidence of the hypothesis that the patients subjectively experience emotional characteristics of their past.

Any behavioral evidence of the hypothesis that the patients possess authentic subjective experiences of their past would be of valuable interest to understand the subjective experience of the patients in the advanced stages of AD. The study supporting the authentic subjective experience hypothesis was conducted in patients with mild AD [16,17]; therefore, research is needed to investigate this experience in patients with advanced AD. This issue is important because advanced AD is characterized by severe language impairment and apathy [52], limiting the ability of the patients to describe memories and their subjective experience. We believe that behavioral studies, such as those on facial expressions, would provide valuable evidence of the hypothesis that patients with advanced AD may possess authentic subjective experiences of their past. Another suggestion for future research would be to examine neural basis of the authentic subjective experience hypothesis. While there is a lack of research on this hypothesis, a research has shown involvement of the hippocampus in the decline of autobiographical memory [53,54]. It would be of interest to investigate whether, despite disruption of the hippocampus, the authentic subjective experience can be supported by other brain regions that are less affected by the disease, at least during the early stages of the AD, such as the visual cortices [55,56].

## 4 Summary

While research on the consciousness experience in AD has been mainly concerned with the compromise of this experience, the authentic subjective experience view attempts to provide an alternative view according to which patients with AD can enjoy, to some extent, an authentic subjective experience during memory retrieval. Although it is important to acknowledge that some components of consciousness experience are affected by AD (e.g., anosognosia), it is equally important to consider whether patients can positively experience other components such as emotion. By doing this, we hope that this narrative review will contribute to a care model that acknowledges and honors the patients’ subjective experiences, provides positive meaning to them, and uses them as a tool in managing the disease.
